# Hemophagocytic lymphohistiocytosis secondary to unrecognized Bartonella henselae infection: a case report

**DOI:** 10.1186/s40794-023-00200-1

**Published:** 2023-09-25

**Authors:** Amanda Hempel, Fizza Manzoor, Dan Petrescu

**Affiliations:** 1 Department of Medicine, University of Toronto, RM 13EN, 300 Elizabeth Street, Toronto, ON M5G 2C4 Canada; 2 Division of Infectious Diseases, University Health Network, Toronto, ON Canada

**Keywords:** Bartonella, Bartonellosis, Cat-scratch disease, Hemophagocytic lymphohistiocytosis, HLH, Immunocompetent, Dog

## Abstract

**Background:**

*Bartonella henselae* is a species of intracellular bacteria transmitted to humans through animal bites and scratches contaminated with the feces of arthropod vectors, and are most commonly associated with cat exposure although transmission from other mammals has been reported. *Bartonella henselae* infection has a spectrum of clinical manifestations and has rarely been reported as cause of hemophagocytic lymphohistiocytosis (HLH) in immunocompromised hosts.

**Case presentation:**

We present a report of *Bartonella henselae* infection progressing to HLH in an immunocompetent patient. The patient initially presented with regional lymphadenopathy but the diagnosis was not suspected as the patient reported no exposure to cats. On further history, he did report a scratch from a dog prior to development of symptoms. The patient was treated with methylprednisolone, intravenous immunoglobulin and anakinra for the HLH and three months of Doxycycline for Bartonella infection, with complete resolution of symptoms.

**Conclusions:**

Although commonly associated with cat exposure, *Bartonella henselae* transmission can occur after exposure to other animals and vectors including dogs and clinicians need to maintain an index of suspicion for timely diagnosis. *Bartonella henselae* is associated with a spectrum of clinical manifestations which can include disseminated infection with severe complications such as hemophagocytic lymphohistiocytosis. Prompt initiation of Bartonella treatment is essential when thought to be the trigger for hemophagocytic lymphohistiocytosis although the optimal treatment regimen is unclear.

## Introduction

*Bartonella henselae* is a species of vector-borne bacteria that has been most commonly associated with direct inoculation through bites and scratches from a cat contaminated with arthropod feces [1, 2]. The association with cats has led to the common name cat-scratch disease, however multiple other animal hosts and arthropod vectors have been implicated in transmission of Bartonella [2–4]. *Bartonella henselae* has a broad spectrum of manifestations, with treatment dependent on the specific presenting syndrome [2]. Hemophagocytic lymphohistiocytosis (HLH) is a life-threatening inflammatory condition that may be precipitated by a variety of infectious triggers including viral, bacterial and fungal pathogens. *Bartonella henselae* has rarely been reported as a cause of HLH, however case reports have occurred in significantly immunocompromised hosts [5–9]. We report here a case of *Bartonella henselae* infection in an immunocompetent patient which was initially not suspected in the absence of cat exposure, that subsequently progressed to HLH requiring emergent treatment.

## Case description

A 25-year-old man presented to hospital with one week of left neck swelling. Four months previously he had recently returned from an eight-month stay in South Africa and his country of birth, Namibia. While in Africa he had stayed with relatives in an urban apartment; he had no animal exposures, health care contact or participation in rural or freshwater activities. His prior medical history included systemic lupus erythematosus (SLE) previously on prednisone and hydroxychloroquine, however he had stopped all immunosuppression over six months prior to presentation out of personal convictions. He also had been incompletely treated for extensively drug-resistant tuberculous meningitis ten years prior.

**Fig. 1:**
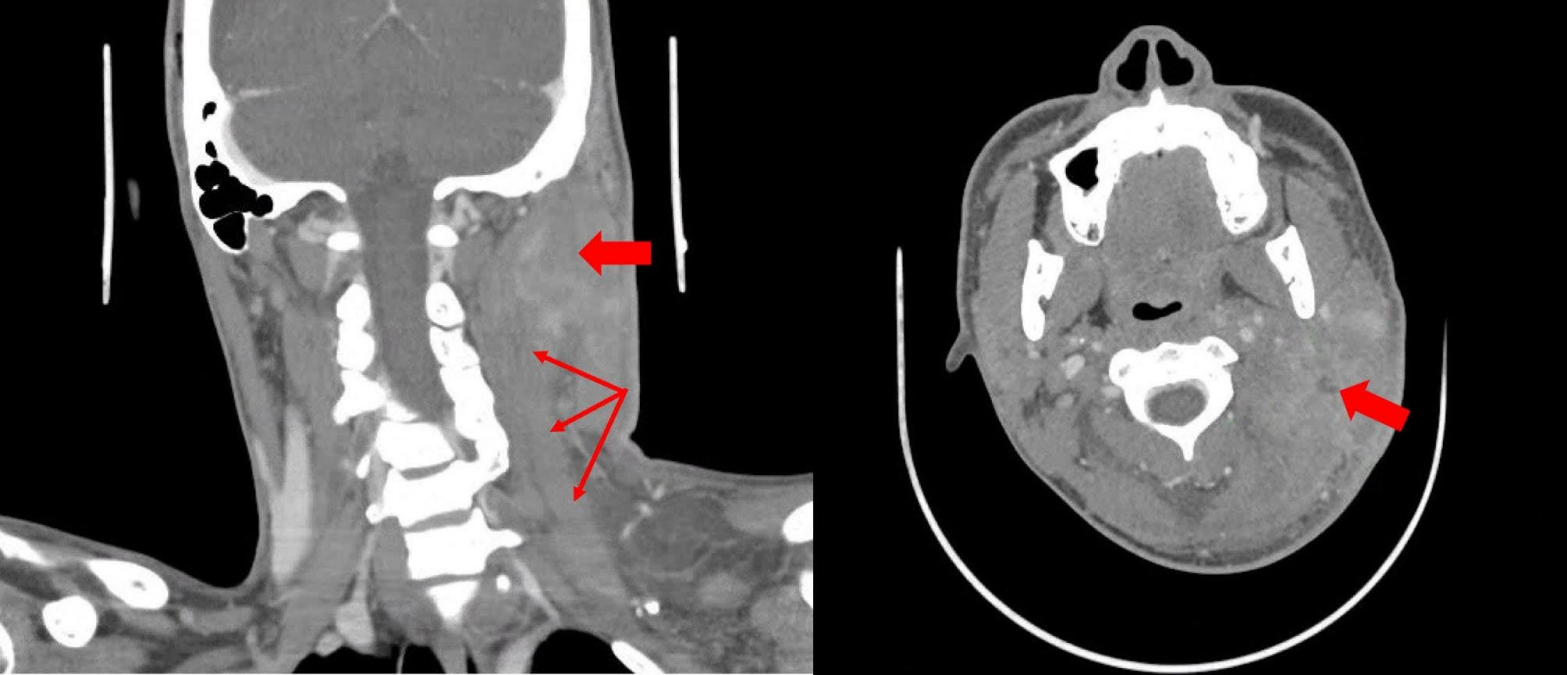
CT Head and Neck demonstrating enhancing soft tissue phlegmon with central necrosis (wide arrows) in the L posterior neck with superimposed myositis of the sternocleidomastoid and paraspinal muscles and soft tissue inflammation, as well as multiple enlarged cervical lymph nodes (thin arrows)

Physical examination revealed soft tissue swelling and warmth of the left neck and earlobe, without fever or other systemic symptoms. A computerized tomography (CT) scan showed necrotic cervical lymphadenopathy with overlying myositis and cellulitis [Fig. 1]. He was assessed by the otolaryngology service which noted cellulitis involving the lower auricle but no tympanic membrane distension or otorrhea. He was discharged on oral amoxicillin-clavulanic acid and ciprofloxacin/dexamethasone ear drops for possible otitis externa.

He returned five days later with worsening pain. Laboratory investigations revealed mild anemia and leukocytosis with normal platelet count and liver enzymes [Table 1]. HIV serology and heterophile antibody were negative. Blood cultures were negative but intravenous piperacillin-tazobactam was initiated for extensive soft tissue infection of the auricle and neck. Given his medical history, there was suspicion that the underlying lymphadenopathy was secondary to mycobacterial lymphadenitis or an SLE flare with secondary cellulitis. *Mycobacterium tuberculosis* DNA was not amplified by polymerase chain reaction (PCR) on fine needle aspirate of the lymph nodes, but there was insufficient tissue for mycobacterial culture or pathology. Excisional biopsy was not possible due to the overlying cellulitis. Blood, urine, and stool samples were smear negative for acid fast bacilli. Serology was not consistent with an SLE flare. Investigations for travel related infections were also sent including negative blood parasite smears and negative serologies for Hepatitis B, Hepatitis C, and Schistosoma. He did have positive strongyloidiasis serology with negative stool ova and parasite examination and was treated with two doses of ivermectin. He was discharged after nine days with a plan to complete fourteen days of piperacillin-tazobactam for the cellulitis followed by an excisional biopsy. Fungal cultures as well as *Bartonella henselae* and *Brucella* serology were pending at the time of discharge.

The patient returned to hospital six days later with fever, sore throat, malaise, and fatigue. The cellulitis had improved. Investigations revealed new pancytopenia, elevated liver enzymes, hypertriglyceridemia, hyperferritinemia, hypofibrinogenemia, and elevated C-reactive protein [Table 1] and a bone marrow biopsy showed hemophagocytosis without signs of lymphoma. He was diagnosed with hemophagocytic lymphohistiocytosis based on the HLH-2004 criteria [Table 2]. Repeat bacterial and fungal blood cultures, Epstein-Barr virus and Cytomegalovirus PCR on blood were negative, as were *Brucella* and histoplasma serologies from his first admission. However, *Bartonella henselae* Immunoglobulin G was noted to be strongly positive at 1:1024. Based on the strongly positive serology and hallmark clinical syndrome of painful regional lymphadenopathy, a presumptive diagnosis of *Bartonella henselae* infection was made. Unfortunately, the lymph node aspirate did not have sufficient sample for PCR or culture.

**Table 1:** Laboratory Investigations on Initial Presentations

	Reference Value	Initial Emergency Department Visit	First Admission	Second Admission
Hemoglobin (g/L)	130–170	112	108	95
Leukocyte Count (10^9^ cells/L)	4–11	11.02	14.06	1.07
Absolute Neutrophil Count (10^9^ cells/L)	2-6.3	6.33	89.93	0.2
Platelet Count (10^9^ cells/L)	140–400	286	3374	49
Creatinine (mmol/L)	42–112	42	39	64
Aspartate Aminotransferase (U/L)	7–40	22	16	775
Alanine Aminotransferase (U/L)	10–45	10	7	283
Alkaline Phosphatase (U/L)	35–125	48	46	59
Bilirubin (µmol/L)	0–23	3	-	3
Ferritin (µg/L)	30–280	103	-	> 67 000
Triglycerides (mmol/L)	< 1.6	-	-	3.34
Fibrinogen (g/L)	1.8-4.00	-	-	1.23
C-reactive Protein (mg/L)	0.0–5.0	-	56.6	129

On further history, the patient reported being scratched on the left arm by a family member’s new pet dog approximately 10–14 days prior to the first emergency department visit; the dog was not known to be unwell, but the patient did not know if it was up to date on vaccinations. The patient had no exposure to cats, rodents or other animals, and had no lice, mites or other known arthropod bites, and had participated in no activities high risk for arthropod bites.

Under multidisciplinary care, he was started on oral doxycycline 100 mg twice daily for *Bartonella henselae* infection, which was presumed to have precipitated HLH. There was initial hesitation to add immunosuppression concern for tuberculous lymphadenitis, however after the negative biopsy and given the progression of HLH, he was ultimately started on high dose methylprednisolone, intravenous immunoglobulin and anakinra. After three days, he began to show clinical improvement and was transitioned to oral prednisone 60 mg daily and other immunosuppressants were stopped. An echocardiogram showed no signs of endocarditis and repeat imagining at six weeks from initial presentation showed resolution of cervical lymphadenopathy. He completed a three-month course of doxycycline and three-month prednisone taper with complete resolution of symptoms.

## Discussion

*Bartonella* are a genus of intracellular bacteria that infect mammalian reservoir hosts and are typically transmitted through arthropod vectors [1, 2]. *Bartonella henselae* is the most well described; cats are considered the reservoir host and transmission occurs through the vector *Ctenocephalides felis* or cat flea [3, 4]. *Bartonella henselae may also be transmitted* to humans and other incidental hosts through arthropod bites or via direct inoculation through cat bites and scratches contaminated with arthropod feces [1, 2]. For this reason, *Bartonella henselae* has been referred to as Cat-Scratch disease, and has a spectrum of clinical including fever, regional lymphadenopathy, ocular and neurologic involvement, culture-negative endocarditis, hepatosplenic lesions, and angio-proliferative lesions [1, 2, 10].

However, understanding of *Bartonella* has been rapidly expanding in the past decades. Multiple *Bartonella* species have been identified in a wide variety of mammalian hosts including humans, cats, dogs, rabbits, rodents, horses and other wild animals [2–4, 11]. A number of additional biting arthropods have also been implicated as vectors including fleas, lice, ticks, bed bugs, flies and mites [2–4, 11]. *Bartonella henselae* specifically has been identified both in other animals such as dogs, as well as in a variety of vectors including ticks, however their capacity to transmit infection to humans has been theorized but not confirmed [2, 3]. Nonetheless, there are numerous reports of human *Bartonella henselae* infection occurring in the absence of any cat or cat flea exposure, suggesting that alternative methods of transmission do occur, and dogs have specifically been implicated in a number of cases [3, 4, 11]. Similarly, our case did not identify any cat or flea exposure, and the dog scratch was the only likely transmission event identified in the period proceeding symptom onset. This raises both One Health concerns about the potential for zoonotic transmission between humans, arthropods and pets, as well as the potential for missed or delayed diagnoses if clinicians only consider cats as an epidemiologic risk factor [3].

Diagnosis is another area of uncertainty because Bartonella are fastidious organisms that do not grow in typical bacterial cultures [2, 10]. Diagnosis is often made clinically based on exposure history and compatible presentation supported by histology (granuloma with pyogenic abscess) and serology [10]. While direct detection methods including culture and PCR have high specificity, neither is sensitive nor used in routine diagnosis though they may play a greater role in atypical presentations [10]. Serology is the most commonly used diagnostic test with IgG values > 1:256 considered highly suggestive of recent infection; IgM production is usually brief and not routinely tested. However, sensitivity of serology is also limited and there is significant cross-reactivity between *Bartonella* species [2]. In our patient, the diagnosis of *Bartonella henselae* was based on the combination of typical presenting syndrome (painful regional lymphadenopathy), compatible exposure, strongly positive serology and response to appropriate treatment. Nonetheless we cannot rule out the possibility that the infection was caused by another cross-reactive *Bartonella* species [10].

Treatment of human bartonellosis depends on the specific presenting syndrome. Lymphadenitis can be treated supportively or with a short course of azithromycin [1, 10]. Neurologic disease is treated with 4–6 weeks of combination therapy with rifampin and doxycycline, while endocarditis is treated with six weeks of doxycycline and rifampin or gentamicin followed by a further 6 weeks of doxycycline alone [1]. For disseminated infection other than neurologic involvement or endocarditis, there is limited data to guide management although azithromycin and rifampin for two weeks has been suggested [1, 10].

HLH is a life-threatening inflammatory condition where unregulated activation of macrophages results in cytokine storm, tissue damage and organ failure [12]. A diagnosis of HLH is made using the HLH-2004molecular or clinical criteria [Table 2] [13, 14]. The three components of HLH treatment include supportive care, treatment/removal of trigger and immunosuppression [13, 14].

HLH is classified as genetic (primary) or acquired (secondary) [12, 14]. Acquired causes of HLH are extensive and include infection, autoimmune disease, malignancy, and medications [14]. Early identification of the trigger for secondary HLH is essential for treatment [12]. Infections are a precipitant in up to half of acquired cases, and in some cases treatment of the underlying infection is sufficient without immunosuppression [14]. Viral infections, particularly Epstein-Barr Virus, are the most common triggers followed by bacterial, fungal and parasitic infections [14]. Bacterial infections constitute 9% of acquired cases of HLH, with the most common bacterial triggers being *Mycobacterium tuberculosis*, *Rickettsia* species, *Staphylococcus* species, and *Escherichia coli.* [14]. The pathophysiology by which such infections trigger HLH is an area of ongoing research.

**Table 2:** HLH-2004 Criteria [13, 15]

Hemophagocytic Lymphohistiocytosis 2004 Criteria		
Molecular Diagnosis	Pathogenic Biallelic Mutation in Genes Associated with HLH	
Clinical and Laboratory Criteria	*At Least 5 of the following*:	
	Fever	
	Splenomegaly	
	Cytopenias	≥ 2 cell lines: • Hemoglobin < 90 g/L • Neutrophils < 1 × 10^9^ cells/L • Platelets < 100 × 10^9^ cells/L
	Hypertriglyceridemia and/or Hyperfibrinogenemia	≥ 3 mmol/L ≤ 1.5 g/L
	Hyperferritinemia	≥ 500 µg/L
	Hemophagocytosis in bone marrow, spleen or lymph nodes without evidence of malignancy	
	Reduced or absent Natural Killer cell activity	
	Elevated soluble CD25 (soluble IL-2 receptor)	≥ 2400 U/mL

*Bartonella* species have been uncommonly reported as a precipitant of HLH; however, the spectrum of clinical presentations of *Bartonella* is still being elucidated. It is hypothesized that intracellular bacteria may trigger HLH because they reside in host immune cells and may continuously activate immune receptors [13]. There are only five published reports linking HLH with *Bartonella* infection, all with *Bartonella henselae* [5–9]. All but one of these cases occurred in immunocompromised hosts and at least three presented initially with lymphadenopathy [5–9]. The majority of the cases had been symptomatic for a prolonged period without diagnosis before presenting in HLH, suggesting a missed opportunity for diagnosis prior to the development of complications [5, 7−9]. Three of the cases had a confirmed history of exposure to cats, but this was not reported for the other cases [5, 7, 8].

In each of the four cases that described management, *Bartonella* was treated with prolonged courses of either doxycycline or combination therapy regardless of clinical syndrome suggesting that the development of HLH was considered sufficient evidence of disseminated infection by the treating clinicians even in the absence of other findings [5, 7−9]. However the exact treatment regimens differed, particularly duration of therapy which varied from 1 month to 1 year. In all cases, immunosuppression for HLH was also required.

The case we present here is unusual in that the patient did not have profound immunosuppression. Although he had a diagnosis of SLE, he had been off immunosuppression for nearly six months at the time of diagnosis. Only one prior case of *Bartonella henselae* leading to HLH in an immunocompetent patient has been reported, in which the patient had two genetic variants associated with susceptibility to HLH [8]. However, it is possible that *Bartonella* has been underdiagnosed given evolving knowledge of risk factors and diagnostic testing discussed here. Given that *Bartonella* frequently present alongside other co-infections, it may also be missed if other serologies are incidentally positive. This may be especially relevant since Bartonella has effective treatments while many viral triggers do not.

Although management of HLH requires prompt initiation of treatment for infection, it is unclear how the development of HLH affects the treatment when other clinical manifestations would typically require only short courses of antibiotics. Despite lymphadenitis being the main presenting symptom in our patient, we elected to consider the infection disseminated as had been done in other reported cases. There is minimal evidence to guide treatment for disseminated bartonellosis and only case reports were available regarding treatment in the setting of HLH.

## Conclusion

Clinicians should be aware of alternative modes of *Bartonella henselae* transmission and should keep a high index of suspicion in the setting of a compatible syndrome even in the absence of cat exposure. They should also be aware of the possibility of dissemination and severe complications such as HLH in untreated cat scratch disease. When *Bartonella* is the suspected trigger of HLH, prompt initiation of treatment is essential however there remains a great deal of uncertainty around what the optimal treatment regimen is in this setting.

## Data Availability

Not applicable.

## List of Abbreviations

CT: Computerized Tomography
HLH: Hemophagocytic Lymphohistiocytosis
PCR: Polymerase Chain Reaction
SLE: Systemic Lupus Erythematosus
